# Association of factors with childhood asthma and allergic diseases using latent class analysis

**DOI:** 10.1038/s41598-024-56805-9

**Published:** 2024-03-22

**Authors:** Teresa To, Cornelia M. Borkhoff, Laura N. Anderson, Catherine S. Birken, Sharon D. Dell, Magdalena Janus, Jonathon L. Maguire, Theo J. Moraes, Patricia C. Parkin, Padmaja Subbarao, Anne Van Dam, Beverly Guttman, Emilie Terebessy, Kimball Zhang, Jingqin Zhu

**Affiliations:** 1 Child Health Evaluative Sciences, Peter Gilgan Centre for Research and Learning, Hospital for Sick Children, 686 Bay St, Toronto, ON M5G 0A4 Canada; 2 Division of Paediatric Medicine and the Paediatric Outcomes Research Team (PORT), Hospital for Sick Children, Toronto, ON Canada; 3 Health Research Methods, Evidence, and Impact, McMaster University, Hamilton, ON Canada; 4 Division of Respiratory Medicine, Department of Pediatrics, BC Children’s Hospital and University of British Columbia, Vancouver, BC Canada; 5 Offord Centre for Child Studies, Department of Psychiatry and Behavioural Neurosciences, McMaster University, Hamilton, ON Canada; 6 The Centre for Urban Health Solutions, Li Ka Shing Knowledge Institute of St. Michael’s Hospital, Toronto, ON Canada; 7 Department of Pediatrics, Translational Medicine, Hospital for Sick Children and University of Toronto, Toronto, ON Canada; 8 Knowledge Mobilization, Canadian Thoracic Society, Ottawa, ON Canada; 9 Provincial Council for Maternal and Child Health, Toronto, ON Canada

**Keywords:** Latent class analysis, Child health determinants, Child health outcomes, Asthma, Allergic disease, Risk factors, Asthma

## Abstract

We hypothesize that children characterized by deprived factors have poorer health outcomes. We aim to identify clustering of determinants and estimate risk of early childhood diseases. This 1993–2019 longitudinal cohort study combines three Canadian pediatric cohorts and their families. Mothers and children are clustered using latent class analysis (LCA) by 16 indicators in three domains (maternal and newborn; socioeconomic status [SES] and neighbourhood; environmental exposures). Hazard ratios (HR) of childhood asthma, allergic rhinitis (AR), and eczema are quantified with Cox proportional hazard (PH) regression. Rate ratios (RR) of children’s health services use (HSU) are estimated with Poisson regression. Here we report the inclusion of 15,724 mother–child pairs; our LCA identifies four mother-clusters. Classes 1 and 2 mothers are older (30–40 s), non-immigrants with university education, living in high SES neighbourhoods; Class 2 mothers have poorer air quality and less greenspace. Classes 3 and 4 mothers are younger (20–30 s), likely an immigrant/refugee, with high school-to-college education, living in lower SES neighborhoods with poorer air quality and less greenspace. Children’s outcomes differ by Class, in comparison to Class 1. Classes 3 and 4 children have higher risks of asthma (HR 1.24, 95% CI 1.11–1.37 and HR 1.39, 95% CI 1.22–1.59, respectively), and similar higher risks of AR and eczema. Children with AR in Class 3 have 20% higher all-cause physician visits (RR = 1.20, 95% CI 1.10–1.30) and those with eczema have 18% higher all-cause emergency department visits (RR = 1.18, 95% CI 1.09–1.28) and 14% higher all-cause physician visits (RR = 1.14, 95% CI 1.09–1.19). Multifactorial-LCA mother-clusters may characterize associations of children’s health outcomes and care, adjusting for interrelationships.

## Introduction

Asthma, allergic rhinitis (AR), and eczema are common childhood inflammatory diseases with multiple risk factors. The epidemiology and etiology of asthma development is complex^1^. The risk of asthma development is multifactorial including genetic, behavioral, and environmental factors (indoor and outdoor exposure to air pollution)^2–4^. For example, a recent study using latent class analysis (LCA) identified five clusters of children from the Danish National Birth Cohort who shared similar patterns of exposure to indoor pollutant sources^3^. While the study uncovered few factors, it found that adolescents growing up in homes with mold during mid-childhood might be at increased odds of current asthma at age 18.

Despite research suggestions of multifactorial interrelationships between maternal, newborn, socioeconomic status (SES), neighbourhood, and environmental determinants of childhood asthma, AR, and eczema, there remains a knowledge gap on the matter in the Canadian population^5,6^. In Canada, clustered profiles of mother–child pairs using LCA that incorporate multifactorial determinants and comprehensive linked health care data have not been constructed, nor used in studying risks and outcomes of common childhood inflammatory diseases and their health services utilization (HSU). This knowledge gap impedes our ability to quantify disease and morbidity risks to prevent poor health outcomes in groups of mother–child pairs who share similar characteristics. Specifically, clustered profiles of multifactorial determinants may better demonstrate which groups of mother–child pairs may be at higher risk of adverse health outcomes.

This study aims to use LCA to incorporate combinations (clusters) of multiple indicators such as maternal, newborn, and SES data collected in three Canadian pediatric cohorts, as well as neighbourhood factors and environmental exposures, to identify distinct profiles and to evaluate their relationships with children’s health outcomes and HSU from health administrative databases (HAD). Identifying clusters of individuals may be useful for public health interventions aimed at preventing the development of asthma and/or allergic diseases in early childhood and adolescence.

## Methods

### Study design and population

We used a longitudinal birth cohort called “FActors of Mothers and Infants in Longitudinal Years” (FAMILY). FAMILY merged children from three pediatric cohorts, as well as their mothers and siblings who were identified through HAD housed at ICES (formerly the Institute for Clinical Evaluative Sciences). The pediatric cohorts were: Toronto site of the Canadian Healthy Infant Longitudinal Development (CHILD) study^7^; The Applied Research Group for Kids (TARGet Kids!)^8^; Toronto Child Health Evaluation Questionnaire (T-CHEQ) study^9^. The CHILD cohort, a national general population-based birth cohort, was established in 2008 to increase our understanding of the interactions between the environment and genetics in the development of asthma and allergy and potentially other common chronic diseases. Since 2008, CHILD enrolled 3624 pregnant mothers (aged > 18 years) from the general population in four major cities across Canada (Vancouver, Edmonton, Winnipeg, and Toronto). The T-CHEQ study was a multistage, cross-sectional study designed to collect population-based prevalence data regarding asthma, other allergic diseases, and possible associated risk factors in Toronto school children attending grades 1 and 2. A total of 5619 grades one and two (aged 5–9 years) Toronto school children were recruited in 2006. TARGet Kids! is an ongoing open longitudinal cohort study enrolling healthy children (from birth to 5 years of age) and following them into adolescence. The aim of the TARGet Kids! cohort is to link early life exposures to health problems including obesity, micronutrient deficiencies, and developmental problems. Children are enrolled during regularly scheduled well-child visits. Once FAMILY was assembled, individuals were linked to HAD to examine childhood disease status and HSU.

There were three inclusion criteria for this study. This study included children of mothers recruited during pregnancy into CHILD, children aged 6–9 years recruited during grades one and two for T-CHEQ, and children under 6 years who were recruited into TARGet Kids! during routine health visits with their primary care physician. Exclusion criteria for this study were: children and mothers (1) without a valid Ontario health card number for data linkage, (2) without an Ontario residence code, (3) who moved away from Ontario during the pregnancy and delivery period, (4) who were missing from the Registered Persons Database (RPDB), (5) missing data in any covariates, and (6) who were missing from the MOMBABY Database. Exclusions also applied to children who (7) died < 28 days after birth, (8) were born after April 2018, and (9) were part of multiple births. See Supplementary Figure S1 for the cohort selection flowchart.

### Data sources

Participants from CHILD, TARGet Kids!, and T-CHEQ were linked to eight Ontario HAD at ICES from the earliest date of birth (1993) to the latest data available (2019). See Supplementary Table S1 for detailed descriptions on HAD used in this study.

This study also used latest available environmental exposure data, namely air pollution, including 2002–2015 ozone (O_3_)^10,11^, 1984–2016 nitrogen dioxide (NO_2_)^12^, 2000–2016 fine particulate matter (PM_2.5_)^11,13^, and urban environment data like greenness measured using the Normalized Difference Vegetation Index (NDVI) available from 1996–2011 and 2013–2015. These data were from the Canadian Urban Environmental Health Research Consortium (CANUE), accessible at ICES. Air pollution and greenness levels were assigned based on postal code at the time of children’s birth using the closest year of available data.

### Covariates and outcomes

We separated covariates into two: concomitant and exposure variables. Concomitant variables were used in LCA to determine class membership, while exposure variables that are known risk factors were included regression analyses for risk prediction. The concomitant variables were selected based on availability in the three study cohorts, health administrative databases, and suggestions from literature.

For concomitant variables, the LCA included 16 factors available from cohorts and/or HAD. *Maternal factors (5)*: age at delivery, highest education attained, immigration status (non-immigrant, landed immigrant, or refugee), pregnancy complications (including hypertension, diabetes, and pre-eclampsia), and no prenatal care visits. *Newborn factors (2):* child ever breastfed and number of siblings in the household at the time of newborn’s birth. *SES and neighbourhood characteristics (4):* SES proxy measured with the Ontario Marginalization Index at neighbourhood-level quintiles (ON-Marg, 4 domains: material deprivation, dependency, ethnic concentration, and residential instability). In this study, those who live in areas with deprivation quintiles 4 or 5, 3, and 1 or 2 are considered as “lower” SES, “average”, and “higher” SES, respectively. *Environmental exposures (5):* air pollution (NO_2_, O_3_, PM_2.5_) and greenness (NDVI) levels at birth year, and indoor environmental tobacco smoke (ETS) exposure.

**Figure 1:**
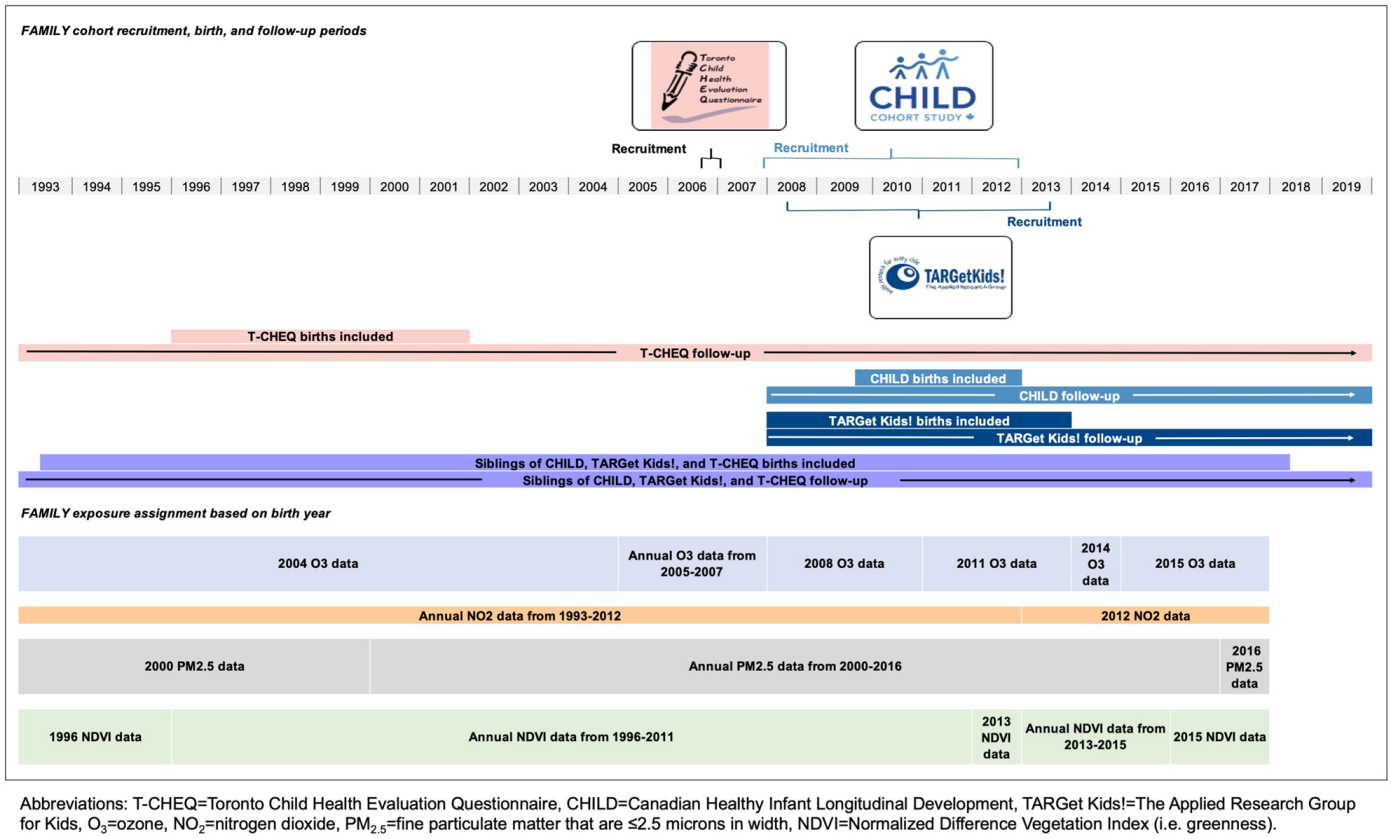
FAMILY cohort recruitment, birth, follow-up, and exposure timelines throughout the study period from 1993 to 2019.

Seven exposure variables were incorporated in regression analyses to measure associations with disease incidence and HSU: “Class” variable generated from the LCA; maternal history of asthma; C-section delivery; birth year; child’s sex; admission to neonatal intensive care unit (NICU) at birth; and low birthweight (< 2500 g). See Fig. 1 for timelines on exposure assignment.

This study examined several outcomes. Incidence of childhood asthma, AR, and eczema were identified from encounters in the Canadian Institute for Health Information Discharge Abstract Database (CIHI-DAD) for hospitalizations, National Ambulatory Care Reporting System (NACRS) for emergency department (ED) visits, and Ontario Health Insurance Plan (OHIP) database for physician office visits using validated health administrative data definitions with International Classification of Diseases (ICD) codes. Children were classified as having incident asthma (ICD-9: 493 and ICD-10: J45, J46) if they had ≥ 2 asthma-related ambulatory care claims in two years or ≥ 1 asthma-related hospitalizations. This validated definition demonstrated 89% sensitivity and 72% specificity in children (0–17 years)^14^. AR (ICD-9: 477 and ICD-10: J301–J304) and eczema (ICD-9: 691.8 and ICD-10: L20) were identified by any physician health services claim for these conditions. All-cause, asthma-specific, and respiratory-related HSU including hospitalizations, ED visits, and physician visits were captured from birth to 2019.

### Statistical analysis

Firstly, we used LCA, a finite mixture model to generate distinct groups of mothers (latent classes) on the basis of similar responses to concomitant variables of interest^15^. 16 variables were included in the LCA: five maternal and two newborn factors, four SES and neighbourhood characteristics, and five environmental. Latent class assignment was made according to the model-based highest membership probability^16^. We evaluated possible models ranging from one to four latent classes. Model fit and selection were determined using BIC (Bayesian Information Criterion), AIC (Akaike Information Criterion), G^2^ (likelihood-ratio chi-square test), Entropy (cut-off > 0.80), Log Likelihood, LMR test (Lo-Mendell-Rubin), as well as calculated model-based predicted probability of membership (Supplementary Table S2).

Next, the latent classes were included in a marginal Cox proportional hazard (PH) regression model to estimate the latent classes-specific hazard ratios (HR) for risk of asthma, AR, and eczema in children. Additionally, six exposure variables were included in the marginal Cox-PH model as covariates. All HR with 95% confidence intervals (CI) were estimated using the marginal Cox PH regression model which accounted for clustering of mother and offspring.

The latent classes were then also included in a Poisson regression model with generalized estimating equations (GEE) to account for the correlation of the responses within family and to estimate the latent classes-specific rate ratios (RR) of all-cause, asthma-specific, and respiratory-related HSU, which are robust to misspecification of the correlation structure. Poisson regressions were stratified by the three childhood disease groups and all RRs were presented with 95% CI. Both Cox PH and Poisson regression models were adjusted for additional potential confounders. These include maternal history of asthma, C-section delivery, birth year, male sex, NICU admission at birth, and low birthweight. We explored possible interactions of covariates with the latent classes, but none were found statistically significant after adjusting for multiple comparisons.

The LCA was conducted using RStudio, poLCA statistical software version 0.98.1091 (R Foundation for Statistical Computing)^15^. All regression analyses were conducted using SAS Enterprise Guide 7.1 (SAS Institute Inc., Cary, NC, USA).

### Research ethics

Ethics approval was obtained from the Hospital for Sick Children Research Ethics Board (Toronto, Ontario, Canada). Informed consent to use cohort participants’ health card numbers to link the study data to health administrative databases was obtained from parents at recruitment in all pediatric cohort studies. ICES is a prescribed entity under section 45 of Ontario’s Personal Health Information Protection Act. Section 45 is the provision that enables ICES to conduct analysis related to the management, evaluation, and monitoring of the health system. Section 45 authorizes health information custodians—like physicians, hospitals, and long-term care homes—to disclose personal health information to a prescribed entity, like ICES, without consent for such purposes.

## Results

### Population characteristics

FAMILY included 15,724 children and their respective mothers. All children in FAMILY were followed in HAD from birth until 2019, an average of 23 years of follow-up. Table 1 shows the distribution of child, maternal, SES, and environmental factors, and disease incidence. The incidence of asthma, AR, and eczema were 22.2% (3492), 25.0% (3932), and 63.1% (9923), respectively. Mothers’ median age at time of delivery was 33 years (interquartile range [IQR]: 30–36), 22.1% (3476) were landed immigrants, and 2.9% (456) were refugees. Nearly 60.0% (9282) of the mothers had university and above education, 93.6% (14,713) reported breastfeeding their babies, 52.8% (8298) of children had no siblings, 39.4% (6189) resided in less deprived neighborhoods (i.e. two lowest quintiles of ON-Marg’s deprivation index) with average levels of air quality and greenspace. The distributions of birth years differed across latent classes in Table 1. This may be due to time of participants’ cohort recruitment; we adjusted for birth year in all regression models to account for potential birth cohort and period effects.

**Table 1:** Percent distributions of characteristics and outcomes of participants by latent class.

Characteristics	Class 1	Class 2	Class 3	Class 4	Total
	% (N = 4835)	% (N = 4161)	% (N = 3683)	% (N = 3045)	% (N = 15,724)
**Concomitant variables for class determination**					
* Maternal and child characteristics*					
Mother's age at delivery					
12–19	0.2	0.2	1.0	4.6	1.2
20–29	7.2	11.7	28.0	43.6	20.3
30–39	82.9	82.0	64.6	47.9	71.6
40–52	9.6	6.0	6.4	3.8	6.8
Mother's highest education attainment					
University and above	78.7	66.5	54.3	23.4	59.0
College	16.8	27.5	28.2	38.0	26.4
High school and below	4.6	6.1	17.5	38.6	14.6
Mother's immigration status					
Non-immigrant	88.6	93.0	51.9	56.7	75.0
Landed immigrant	10.6	6.8	42.3	36.8	22.1
Refugee	0.8	0.2	5.7	6.5	2.9
Prevalence of pregnancy complications (pregnancy induced hypertension, gestational diabetes, pre-eclampsia)	5.1	4.8	6.6	8.6	6.1
No prenatal care visit during pregnancy	7.1	7.8	4.6	2.9	5.9
Child being breastfed	96.9	96.1	92.6	85.9	93.6
Number of siblings in the household when the child was born					
0	44.4	57.9	49.9	62.6	52.8
1	42.7	34.8	34.2	28.3	35.8
2	11.2	6.4	11.3	7.3	9.2
≥ 3	1.7	0.9	4.6	1.8	2.2
*Neighbourhood characteristics (Ontario Marginalization Index)*					
Deprivation quintiles (Q1–Q5)					
Q1 (least)	32.4	40.2	9.9	3.0	23.5
Q2	32.4	25.2	8.3	5.4	19.6
Q3	21.1	19.0	14.7	13.4	17.5
Q4	11.6	13.4	27.2	24.8	18.3
Q5 (most)	2.5	2.3	39.9	53.4	21.1
Instability quintiles (Q1–5)					
Q1 (least)	9.1	4.4	20.3	3.3	9.3
Q2	12.3	10.8	10.9	6.1	10.4
Q3	20.7	18.7	9.8	13.3	16.2
Q4	31.1	32.0	14.4	32.5	27.7
Q5 (most)	26.7	34.1	44.6	44.8	36.4
Ethnic concentration quintiles					
Q1 (least)	1.8	8.3	0.4	0.0	2.8
Q2	10.2	20.5	0.0	0.3	8.6
Q3	40.9	35.0	0.1	2.4	22.3
Q4	47.0	31.7	18.0	22.9	31.5
Q5 (most)	0.1	4.3	81.5	74.4	34.7
Dependency quintiles					
Q1 (least)	28.2	23.3	51.0	28.4	32.3
Q2	30.2	26.2	24.5	27.0	27.2
Q3	20.7	21.3	11.8	21.3	18.9
Q4	11.8	15.9	7.9	14.4	12.5
Q5 (most)	9.1	13.3	4.8	8.8	9.1
* Environmental exposures*					
Environmental tobacco smoke exposure	8.2	4.7	16.9	21.1	11.8
Nitrogen dioxide quartiles (Q1–4)					
Q1: ≤ 16.3	50.7	0.3	41.8	0.2	25.5
Q2: > 16.3–≤ 19.9	44.6	6.4	38.6	3.1	25.1
Q3: > 19.9–≤ 24.4	4.8	49.1	17.2	31.3	24.5
Q4: > 24.4	0.0	44.2	2.4	65.4	24.9
Ozone					
Q1: ≤ 19.61	0.0	56.1	0.2	60.6	26.6
Q2: > 19.61–≤ 22.55	30.9	18.4	25.8	22.4	24.8
Q3: > 22.55–≤ 24.03	38.1	11.3	34.8	8.8	24.5
Q4: > 24.03	31.0	14.2	39.3	8.3	24.1
Fine particulate matter ≤ 2.5 μm in width quartiles (Q1–4)					
Q1: ≤ 7.9	46.2	0.9	46.1	4.1	26.0
Q2: > 7.9–≤ 8.8	33.0	12.5	32.3	16.9	24.3
Q3: > 8.8–≤ 9.5	18.1	31.7	17.1	36.4	25.0
Q4: > 9.5	2.7	54.8	4.4	42.7	24.6
Normalized Difference Vegetation Index					
Q1: ≤ 0.254	13.8	17.0	35.7	41.6	25.2
Q2: > 0.254–≤ 0.343	18.6	22.1	31.5	32.5	25.2
Q3: > 0.343–≤ 0.429	28.5	29.9	19.9	17.4	24.7
Q4: > 0.429	39.1	31.0	12.9	8.5	24.9
**Predictor variables**					
Maternal history of asthma	17.2	16.7	15.7	19.1	17.1
C-section delivery	29.0	25.6	29.3	21.6	26.7
Male sex	52.1	51.2	52.8	51.7	51.9
Birth year					
1993–1999	1.5	29.8	2.5	40.3	16.7
2000–2004	1.1	28.1	2.2	28.0	13.7
2005–2009	19.8	38.9	22.8	28.2	27.2
2010–2014	52.1	1.9	50.9	2.8	29.0
2015–2017	25.5	1.2	21.6	0.7	13.3
Admission to neonatal intensive care unit at birth	9.2	9.7	13.6	14.0	11.3
Baby born with low birth weight	4.7	4.1	8.0	6.4	5.6
**Outcomes**					
Asthma	16.9	23.0	21.4	30.5	22.2
Allergic rhinitis	14.9	30.4	19.7	40.0	25.0
Eczema	55.3	66.7	60.0	74.4	63.1

### Latent Class Identification

**Figure 2:**
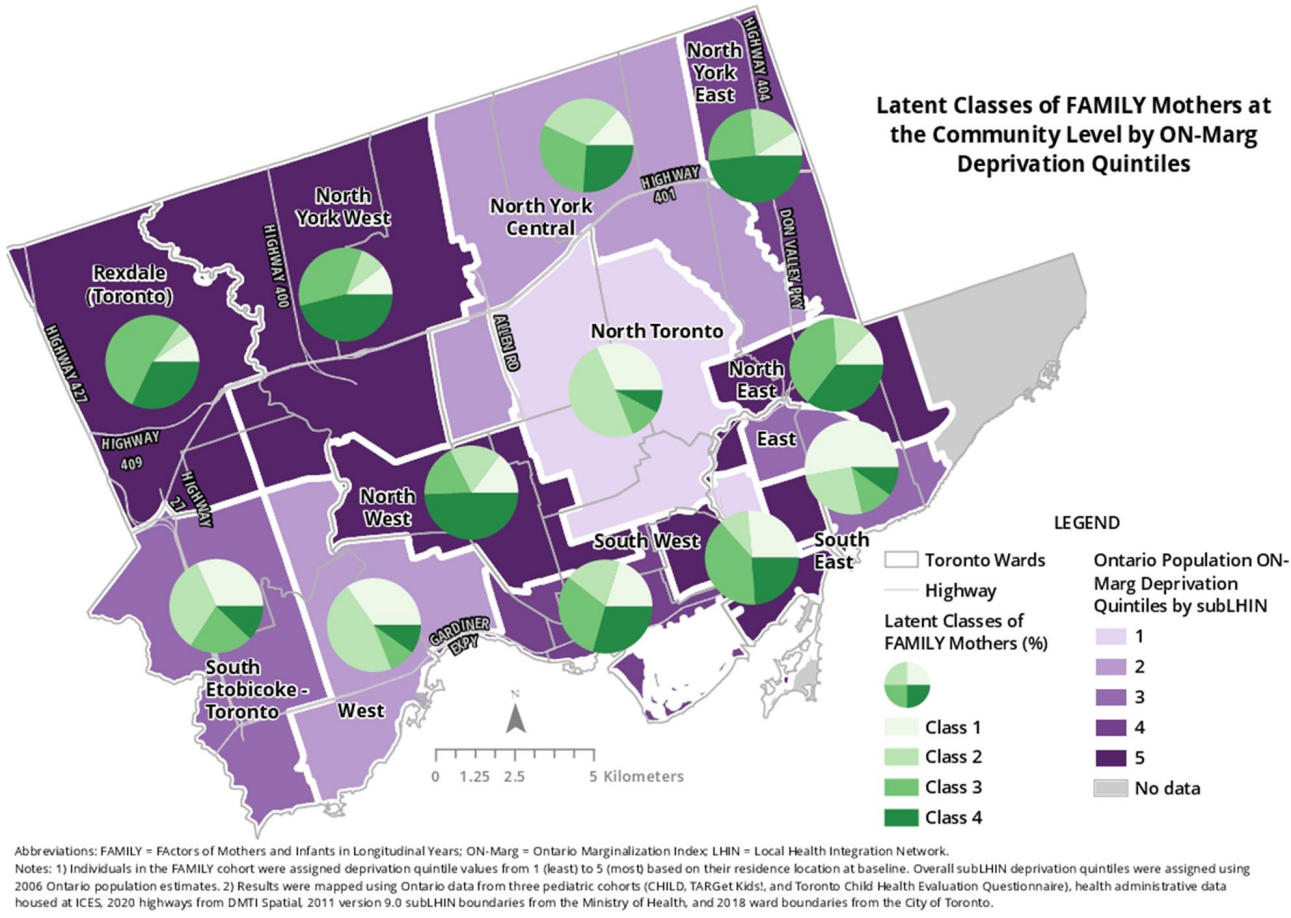
Geographical distribution of latent classes in the Greater Toronto Area in Ontario, Canada.

16 concomitant variables listed in Table 1 were included in the analysis to determine latent class membership. Supplementary Table S2 shows goodness-of-fit statistics of the latent class models. We used the 4-class model as its fit statistics were best (lowest AIC, BIC, G^2^, and highest Entropy). The 4-class model was labeled/named based on key characteristics. The following characteristics are found in a typical member of their respective class. For example, Class 1: *mothers in their 30 s–40 s* with university or above education, non-immigrants, who likely had one or two children, lived in high SES neighborhoods with good air quality and greenspace. Class 2: *mothers who were* > *30 s* with university or above education, non-immigrants, who likely had a single child, lived in a high SES neighborhood, but with relatively poorer air quality and lower amount of greenspace. Class 3: *mothers in their 30 s* with university or above education, likely a landed immigrant (or a refugee), with one or more children, lived in average SES neighborhoods with relatively good air quality and a good amount of greenspace. Class 4: *mothers in their 20 s* with high school to college education, likely a landed immigrant (or a refugee) and with a single child, lived in lower SES neighborhoods with high traffic-related air pollution and lower amount of greenspace. Table 1 and Fig. 2 show the distribution of covariates and geographic distribution of the four latent classes, respectively. Supplementary Table S3 and Supplementary Figure S2 show the distribution of conditional probabilities by covariates and latent classes.

### Association of disease incidence risk–Cox proportional hazard (PH) regression

Results from the multivariable Cox PH regression analyses (Table 2) showed that children born to mothers with maternal history of asthma had significantly increased risks of asthma, AR, and eczema. Children who were born by C-section (HR 1.10, 95% CI 1.02–1.19), with low birthweight (HR 1.31, 95% CI 1.14–1.51), being male (HR 1.42, 95% CI 1.33–1.52), and whose mother had a history of asthma (HR 1.64, 95% CI 1.50–1.79) had significantly higher risks for asthma. Children who were admitted to the NICU at birth also had significantly increased risk for asthma (HR 1.35, 95% CI 1.22–1.50), but not for AR nor eczema. Overall, similar findings were seen in AR and eczema, albeit the risks were lower with wider confidence intervals. After adjusting for all exposure variables, results showed that compared to children in Class 1, children in Classes 3 and 4 had higher risks of asthma (HR 1.24, 95% CI 1.11–1.37 and HR 1.39, 95% CI 1.22–1.59, respectively). Children in Classes 3 and 4 also had higher risks of AR (HR 1.26, 95% CI 1.14–1.41 and HR 1.39, 95% CI 1.23–1.57, respectively) and eczema (HR 1.12, 95% CI 1.06–1.19 and HR 1.15, 95% CI 1.06–1.24, respectively) compared to those in Class 1.

**Table 2:** Hazard ratios from Cox PH regression analyses adjusted for latent classes and exposure variables for asthma, allergic rhinitis, and eczema.

Covariates	Asthma		Allergic rhinitis		Eczema	
	Hazard ratio (HR)	95% confidence interval (CI)	HR	95% CI	HR	95% CI
Class 2 (reference: Class 1)	1.06	0.93–1.20	1.04	0.93–1.18	0.93	0.86–1.00
Class 3	1.24	1.11–1.37	1.26	1.14–1.41	1.12	1.06–1.19
Class 4	1.39	1.22–1.59	1.39	1.23–1.57	1.15	1.06–1.24
Maternal history of asthma	1.64	1.50–1.79	1.34	1.23–1.46	1.15	1.09–1.22
C-section delivery	1.10	1.02–1.19	1.01	0.94–1.09	1.05	1.00–1.11
Male sex (reference: female)	1.42	1.33–1.52	1.27	1.19–1.36	0.99	0.95–1.03
Birth year (reference: 1993–1999)						
2000–2004	0.79	0.70–0.88	0.81	0.74–0.89	0.85	0.79–0.91
2005–2009	0.77	0.70–0.86	0.76	0.69–0.84	0.72	0.68–0.77
2010–2014	0.79	0.68–0.91	0.76	0.66–0.87	0.74	0.68–0.80
2015–2017	0.83	0.70–0.98	0.68	0.56–0.83	0.88	0.80–0.97
Admission to neonatal intensive care unit at birth	1.35	1.22–1.50	1.10	0.99–1.22	1.01	0.94–1.09
Baby born with low birth weight	1.31	1.14–1.51	0.98	0.85–1.14	0.93	0.85–1.02

#### Association of health services utilization (HSU)–Poisson regression

Results from the Poisson regression analyses (Table 3) showed that amongst children with asthma, maternal history of asthma increased children’s risk of asthma-specific and all-cause HSU. Compared to children in Class 1, children with asthma in Class 2 had twofold significantly lower asthma ED visit rates (RR = 0.65, 95% CI 0.47–0.89), and lower all-cause ED visit rates (RR = 0.82, 95% CI 0.70–0.95). Compared to Class 1, children with AR in Class 3 had a significantly higher all-cause physician visit rate (RR = 1.20, 95% CI 1.10–1.30). Similarly, children with eczema in Class 3 had both significantly higher all-cause ED visit rates (RR = 1.18, 95% CI 1.09–1.28) and all-cause physician visit rates (RR = 1.14, 95% CI 1.09–1.19).

**Table 3:** Rate ratios of health services utilization from Poisson regression analyses adjusted for latent classes and exposure variables for asthma, allergic rhinitis, and eczema.

Covariates	Among children with asthma (N = 3492)					
	Asthma-specific					
	Hospitalizations		Emergency department visits		Physician visits	
	Rate ratio (RR)	95% confidence interval (CI)	RR	95% CI	RR	95% CI
Class 2 (reference: Class 1)	0.88	0.40–1.94	0.65	0.47–0.89	0.82	0.67–1.00
Class 3	1.10	0.61–1.96	0.98	0.71–1.36	1.03	0.89–1.19
Class 4	1.89	0.91–3.93	1.15	0.84–1.57	0.90	0.73–1.10
Maternal history of asthma	1.60	1.06–2.41	1.29	1.00–1.67	1.14	1.03–1.27
C-section delivery	1.19	0.83–1.71	0.89	0.69–1.15	0.94	0.86–1.04
Male sex (reference: female)	1.03	0.75–1.43	1.41	1.11–1.78	1.12	1.02–1.22
Birth year (reference: 1993–1999)						
2000–2004	1.34	0.82–2.19	1.16	0.83–1.62	1.23	1.08–1.40
2005–2009	1.54	0.98–2.43	1.20	0.87–1.65	1.48	1.29–1.70
2010–2014	2.60	1.31–5.13	1.54	1.13–2.10	2.12	1.71–2.61
2015–2017	3.95	1.74–8.99	1.07	0.65–1.77	3.08	2.43–3.91
Admission to neonatal intensive care unit at birth	0.88	0.54–1.43	1.17	0.91–1.52	1.09	0.95–1.26
Baby born with low birth weight	1.64	0.89–3.02	1.40	0.96–2.05	1.12	0.91–1.39

Covariates	Among children with asthma (N = 3492)					
	All-cause					
	Hospitalization		Emergency department visits		Physician visits	
	Rate ratio (RR)	95% confidence interval (CI)	RR	95% CI	RR	95% CI
Class 2 (reference: Class 1)	0.66	0.42–1.03	0.82	0.70–0.95	0.90	0.81–1.00
Class 3	1.02	0.74–1.39	1.06	0.94–1.21	1.09	1.01–1.17
Class 4	1.01	0.64–1.59	0.99	0.84–1.15	0.99	0.90–1.09
Maternal history of asthma	1.43	1.11–1.85	1.31	1.20–1.44	1.12	1.05–1.18
C-section delivery	0.94	0.71–1.25	0.98	0.89–1.07	1.03	0.97–1.09
Male sex (reference: female)	0.86	0.68–1.10	1.12	1.04–1.21	0.95	0.91–1.00
Birth year (reference: 1993–1999)						
2000–2004	1.46	1.11–1.92	1.20	1.06–1.35	0.98	0.92–1.05
2005–2009	1.66	1.18–2.34	1.37	1.23–1.52	0.98	0.91–1.06
2010–2014	1.29	0.87–1.89	1.54	1.32–1.79	1.14	1.04–1.25
2015–2017	2.16	1.38–3.39	1.81	1.48–2.20	1.49	1.33–1.66
Admission to neonatal intensive care unit at birth	1.86	1.33–2.62	1.16	1.04–1.30	1.14	1.04–1.25
Baby born with low birth weight	1.36	0.93–2.00	1.13	0.97–1.32	0.97	0.88–1.08

Covariates	Among children with allergic rhinitis (N = 3932)					
	All-cause					
	Hospitalization		Emergency department (ED) visits		Physician visits	
	Rate ratio (RR)	95% confidence interval (CI)	RR	95% CI	RR	95% CI
Class 2 (reference: Class 1)	0.54	0.28–1.07	0.92	0.77–1.10	0.96	0.86–1.07
Class 3	0.95	0.57–1.59	1.15	0.98–1.34	1.20	1.10–1.30
Class 4	0.51	0.26–1.00	0.95	0.79–1.14	1.02	0.92–1.13
Maternal history of asthma	1.51	1.10–2.06	1.40	1.25–1.56	1.14	1.07–1.22
C-section delivery	0.97	0.75–1.25	0.97	0.87–1.08	1.03	0.96–1.10
Male sex (reference: female)	1.16	0.91–1.48	1.07	0.98–1.17	0.95	0.90–0.99
Birth year (reference: 1993–1999)						
2000–2004	0.84	0.61–1.14	1.09	0.97–1.23	0.95	0.89–1.00
2005–2009	0.98	0.66–1.45	1.23	1.09–1.38	0.98	0.90–1.07
2010–2014	0.79	0.42–1.50	1.28	1.06–1.56	1.16	1.05–1.28
2015–2017	1.44	0.65–3.18	1.89	1.45–2.46	1.53	1.36–1.73
Admission to neonatal intensive care unit at birth	1.38	0.98–1.94	1.14	0.99–1.32	1.11	0.98–1.25
Baby born with low birth weight	1.13	0.70–1.82	1.05	0.85–1.29	1.10	0.94–1.27

Covariates	Among children with eczema (N = 9923)					
	All-cause					
	Hospitalization		ED visits		Physician visits	
	RR	95% CI	RR	95% CI	RR	95% CI
Class 2 (reference: Class 1)	0.68	0.50–0.94	0.93	0.84–1.02	0.96	0.91–1.02
Class 3	1.08	0.84–1.38	1.18	1.09–1.28	1.14	1.09–1.19
Class 4	0.90	0.64–1.26	1.08	0.97–1.20	1.05	1.00–1.12
Maternal history of asthma	1.35	1.11–1.64	1.33	1.24–1.43	1.16	1.12–1.21
C-section delivery	1.02	0.84–1.25	0.99	0.93–1.05	1.05	1.02–1.09
Male sex (reference: female)	1.15	0.97–1.36	1.11	1.06–1.16	1.00	0.98–1.03
Birth year (reference: 1993–1999)						
2000–2004	0.85	0.69–1.04	1.03	0.96–1.11	0.92	0.88–0.96
2005–2009	0.97	0.76–1.23	1.19	1.10–1.28	0.94	0.90–0.99
2010–2014	0.88	0.64–1.23	1.27	1.14–1.41	1.10	1.04–1.17
2015–2017	1.04	0.73–1.50	1.63	1.43–1.86	1.52	1.42–1.62
Admission to neonatal intensive care uni at birth	1.71	1.32–2.22	1.20	1.10–1.30	1.11	1.06–1.17
Baby born with low birth weight	1.15	0.82–1.60	1.05	0.93–1.18	1.03	0.97–1.10

## Discussion

Our study included a large cohort of mother–child pairs with extensive data from multiple sources over a long time. We used LCA, a person-centred technique to identify and elucidate the clustering of risk and atopy related health outcomes^10^ in offspring. The interrelationship between SES, maternal and newborn factors, as well as environmental and neighborhood factors highlight the multifactorial development of common childhood diseases. Compared to children in Class 1, children born to younger mothers in Class 4 (including children from lower SES families residing in more deprived neighborhoods with poorer air quality and less greenspace) had a clinically meaningful 39% higher risk of asthma, AR, and 15% higher risk of eczema. Moreover, children with asthma in Class 2, compared to those in Class 1, had significantly lower asthma ED visit rates. This may be related to lower exposure to O_3_ in Class 2, despite a larger proportion of individuals living in neighborhoods with higher levels of other traffic-related pollution (e.g. NO_2_ and PM_2.5_). This is consistent with those reporting an association of increased asthma ED visits with increased O_3_ exposure^17–22^.

Our findings are consistent with those of previous studies using LCA. Hardie et al. used U.S. data from the National Health Interview Surveys in 2007 and 2008 (N = 7361) to evaluate the joint implications of maternal health and SES disadvantage for youth, and found that children in the low-SES class (like our Class 3) had 43% higher odds of having asthma than the low-risk class (like our Class 1)^23^. Kozyrskyj et al. used data from the Western Australian Pregnancy Cohort (Raine) to study associations between changes in family SES and childhood asthma^24^. Their LCA indicated a twofold increased risk of asthma at age 14 years amongst children who had lived in a low-income family since birth, especially girls. Compared with children in chronic low-income families, children in households with increasing incomes had 60% lower risk of asthma. Grunwell et al. used LCA including 513 children aged 6–17 years at risk for asthma exacerbation and identified four classes according to demographics, sensitization, Type-2 inflammatory markers, and lung functions^25^. They found that those with multiple sensitization with airflow limitation had a higher percentage of parents with high school or below education and had exposure to indoor smoking. Abuabara et al. used the Pediatric Eczema Elective Registry data and LCA to identify patterns of disease control and found that income < $50,000/year was strongly associated with eczema persistence (odds ratio [OR] = 1.69, 95% CI 1.37–2.08)^26^. Shakerkhatibi et al. studied air pollution-related asthma profiles among children and adolescents in Iran using LCA^27^. They observed a higher probability of severe asthma (6.8%) in a case village located in a polluted industrial area compared to control communities (2.6% and 1.8%) with no potential of urban air pollution. Additionally, adjusted odds of asthma were lower in the control communities than the case community in both moderate and severe asthma classes with significant ORs ranging from 0.14–0.70 to 0.32–0.53, in the respective classes. Sbihi et al. used HAD of medical visits from British Columbia, Canada to define the occurrence and recurrence of asthma over a 10-year follow-up period^28^. Instead of LCA, the authors used a group-based trajectory modeling method to identify asthma trajectories. They found that an interquartile increase in exposure to NO_2_ increased the risk of membership in the early and late-onset chronic asthma trajectories and concluded that traffic-related air pollution increased the probability of a chronic asthma trajectory. Our study also identified that Classes 3 and 4 mother–child pairs (higher risk groups) were more likely to cluster in more deprived areas in the northwest region of the Greater Toronto Area (Fig. 1).

Consistent with literature reporting lower incidence of asthma, we also observed that children born in the 2000s, compared to the 1990s, had lower HRs for asthma and other allergic diseases^29,30^. The reasons behind this decrease in high-income countries (e.g., US, Canada, UK, Australia) remain unclear. However, others have hypothesized that improvements in air quality, improved primary care, higher breastfeeding rate, lower antibiotics’ use in infants, etc., may be contributing factors^29,31–35^.

There are limited longitudinal studies that examine how maternal characteristics, SES, neighbourhood, and environmental determinants influence child health outcomes. This study’s greatest strength is addressing this gap with a large longitudinal birth cohort called FAMILY. FAMILY merged three Ontario pediatric cohorts, then used HAD to efficiently identify cohort participants and their mothers, forming Classes of mother–child pairs, as well as participants’ siblings. We included siblings to expand the size of the FAMILY cohort to study child health outcomes among more children beyond the cohort participants, as well as included number of siblings in the household at the time of newborn’s birth as part of the concomitant variable in the LCA. Another strength is the use of HAD spanning decades to efficiently conduct longitudinal analyses with minimal challenges. This study also has limitations. First, we lack self-reported, clinical, and biomarker factors in the FAMILY cohort analyses. This is a consequence of consolidating varying, albeit rich, individual-level information from different pediatric cohorts that used different survey instruments. Another limitation is the lack of access to reliable information from HAD on maternal lifestyle factors, namely smoking or medication use during pregnancy, as well as linked data on fathers. Since we lacked longitudinal status of all variables, we did not use the latent transition analysis model, which could examine the variation over time and to identify the association of repeated measures.

In conclusion, using LCA to identify clusters of mother–child pairs, we found environmental exposures and neighborhood factors that were significantly associated with asthma, allergic disease health outcomes, and HSU while adjusting for multifactorial interrelationships. Findings can be shared with respiratory therapists/educators and primary health care providers to provide education on disease self-management and shared with policy makers to assist families with funding to support medication use. This is particularly important for children with asthma who have high acute care demands as it may be a “marker” of poor access to primary care or poor disease self-management. Our findings also raised awareness about the health risks of environmental exposures (air pollution and lack of greenspace), especially for children with Class 3 and 4 mothers, who have a higher risk of asthma and allergic diseases. Our findings may help stakeholders develop strategies or programs to reduce environmental exposures. Future studies with LCA and linked HAD may include medication and laboratory test utilizations (e.g., eosinophil blood tests) to gain insight on using these factors as predictors in child health outcomes.

### Supplementary Information

Supplementary Information.

## Data Availability

The data that support the findings of this study are available at ICES, Ontario, Canada but restrictions apply to the availability of these data, which were used under license for the current study, and so are not publicly available. Specifically, the dataset from this study is held securely in coded form at ICES. While legal data sharing agreements between ICES and data providers (e.g., healthcare organizations and government) prohibit ICES from making the dataset publicly available, access may be granted to those who meet pre-specified criteria for confidential access, available at https://www.ices.on.ca/use-ices-data/. Contact ICES at the following e-mail for more information on data access: das@ices.on.ca.

## Abbreviations

SES: Socioeconomic status
AR: Allergic rhinitis
HSU: Health services utilization
LCA: Latent class analysis (LCA)
HAD: Health administrative databases
FAMILY: FActors of Mothers and Infants in Longitudinal Years
CHILD: Canadian Healthy Infant Longitudinal Development
TARGet Kids!: The Applied Research Group for Kids
T-CHEQ: Toronto Child Health Evaluation Questionnaire
RPDB: Registered Persons Database
O_3_: Ozone
NO_2_: Nitrogen dioxide
PM_2.5_: Fine particulate matter that are ≤ 2.5 μm in width
NDVI: Normalized Difference Vegetation Index
CANUE: Canadian Urban Environmental Health Research Consortium
ON-Marg: Ontario Marginalization Index
ETS: Environmental tobacco smoke
NICU: Neonatal intensive care unit
CIHI-DAD: Canadian Institute for Health Information Discharge Abstract Database
NACRS: National Ambulatory Care Reporting System
ED: Emergency Department
OHIP: Ontario Health Insurance Plan
ICD: International Statistical Classification of Diseases
BIC: Bayesian Information Criterion
AIC: Akaike Information Criterion
G^2^: Likelihood-ratio chi-square test
LMR: Lo-Mendell-Rubin
PH: Proportional hazard
HR: Hazard ratio
CI: Confidence intervals
RR: Rate ratio
IQR: Interquartile range
OR: Odds ratio
LBW: Low birthweight

## Code availability

The underlying code that supports the analysis and findings of this study, utilized in a secure analysis environment at ICES, Ontario, Canada, is available from the authors upon request. Note that the computer programs may rely upon coding templates or macros that are unique to ICES and are, therefore, either inaccessible or may require modification. Requests to access the datasets should be directed to ICES data sharing, das@ices.on.ca.
