# Supplementary material for: Association of factors with childhood asthma and allergic diseases using latent class analysis
Source: Sci Rep. 2024 Mar 22;14:6874. doi: 10.1038/s41598-024-56805-9 (PMC10959981; doi:10.1038/s41598-024-56805-9)
Supplement: Supplementary file 1 — Supplementary Information. [file 41598_2024_56805_MOESM1_ESM.docx]

**Supplementary Table S1.** Health administrative data sources housed at ICES.

| Number | Database | Information on Data |
| --- | --- | --- |
| 1 | Ontario Health Insurance Plan (OHIP) Database | Contains information on all fee-for-service billings for physician services |
| 2 | Canadian Institute for Health Information Discharge Abstract Database (CIHI-DAD) | Records hospitalizations using the most responsible diagnoses for all patients discharged from acute-care hospitals |
| 3 | National Ambulatory Care Reporting System (NACRS) | Contains data for emergency department (ED) visits, coded using International Classification of Disease (ICD) codes |
| 4 | Registered Persons Database (RPDB) | Includes information on sex, date of birth, and residence postal code |
| 5 | Ontario Marginalization Index (ON-Marg) | ON-Marg uses Census data to measure four dimensions of marginalization (material deprivation, dependency, ethnic concentration, and residential instability) at the neighbourhood-level; individuals in the study were assigned a value from 1 (least marginalized) to 5 (most marginalized) for each dimension based on residence at baseline |
| 6 | Ontario Asthma Surveillance Information System (OASIS; <https://lab.research.sickkids.ca/oasis/>) | OASIS is a cohort of Ontarians aged 0-99 years old with physician-diagnosed asthma between 1996 and 2021 and was used to identify date of asthma diagnosis |
| 7 | Mom-Baby Database | Used to identified mothers and siblings of children in the pediatric cohorts |
| 8 | Permanent Resident Database of Citizenship and Immigration Canada (CIC) | Contains information on Ontario immigrants between 1985-2020 and was used to establish the immigration status of study participants |

**Supplementary Table S2.** Latent class model fit statistics, N=15,724.

|  |  |  |  |  |  |  |  |  | Predicted class membership |  |  |  |
| --- | --- | --- | --- | --- | --- | --- | --- | --- | --- | --- | --- | --- |
| Fit statistics |  |  |  |  |  |  |  |  | by model posterior probability |  |  |  |
| Number of Classes | Maximum Log-likelihood | Residual Degrees of Freedom | Bayesian Information Criterion | Akaike Information Criterion | Likelihood-ratio chi-square test | Entropy | Lo-Mendell-Rubin Test |  | 1 | 2 | 3 | 4 |
| 1 |  | 15682.00 | 502671.50 | 502349.70 | 200833.10 | NA |  |  | 1.0000 |  |  |  |
| 2 | -243656.80 | 15639.00 | 488134.90 | 487483.50 | 185881.00 | 0.85 |  |  | 0.4674 | 0.5320 |  |  |
| 3 | -239259.70 | 15596.00 | 479756.20 | 478775.30 | 177086.80 | 0.81 | p<0.001 |  | 0.3082 | 0.2842 | 0.4076 |  |
| 4 | -236656.50 | 15553.00 | 474965.30 | 473655.00 | 171880.40 | 0.82 | p<0.001 |  | 0.3075 | 0.2646 | 0.2342 | 0.1937 |

**Supplementary Table S3.** Conditional probabilities by concomitant variables and the latent classes, N=15,724.

| Indicator Variables | Class 1 | Class 2 | Class 3 | Class 4 |
| --- | --- | --- | --- | --- |
| Maternal and child characteristics |  |  |  |  |
| Mother’s age at delivery |  |  |  |  |
| 12-19 | 0.0025 | 0.0023 | 0.0090 | 0.0450 |
| 20-29 | 0.0745 | 0.1193 | 0.2696 | 0.4280 |
| 30-39 | 0.8275 | 0.8164 | 0.6573 | 0.4886 |
| 40-52 | 0.0956 | 0.0619 | 0.0642 | 0.0384 |
| Mother’s highest education attainment |  |  |  |  |
| University and above | 0.7858 | 0.6614 | 0.5504 | 0.2499 |
| College | 0.1681 | 0.2760 | 0.2811 | 0.3727 |
| High school and below | 0.0462 | 0.0626 | 0.1685 | 0.3774 |
| Mother’s immigration status |  |  |  |  |
| Non-immigrant | 0.8833 | 0.9251 | 0.5389 | 0.5742 |
| Landed immigrant | 0.1081 | 0.0723 | 0.4063 | 0.3625 |
| Refugee | 0.0086 | 0.0025 | 0.0548 | 0.0633 |
| Prevalence of pregnancy complications (pregnancy induced hypertension, gestational diabetes, pre-eclampsia) | 0.0518 | 0.0484 | 0.0654 | 0.0842 |
| No prenatal care visit during pregnancy | 0.0706 | 0.0770 | 0.0469 | 0.0310 |
| Child being breastfed | 0.9688 | 0.9598 | 0.9296 | 0.8614 |
| Number of siblings in the household when the child was born |  |  |  |  |
| 0 | 0.4432 | 0.5763 | 0.4995 | 0.6264 |
| 1 | 0.4275 | 0.3491 | 0.3442 | 0.2833 |
| 2 | 0.1127 | 0.0658 | 0.1122 | 0.0716 |
| ≥3 | 0.0166 | 0.0088 | 0.0441 | 0.0187 |
| Neighborhood characteristics (Ontario Marginalization Index): |  |  |  |  |
| Deprivation Quintiles (Q) |  |  |  |  |
| Q1 (Least) | 0.3283 | 0.3983 | 0.1030 | 0.0396 |
| Q2 | 0.3242 | 0.2523 | 0.0883 | 0.0593 |
| Q3 | 0.2071 | 0.1882 | 0.1544 | 0.1358 |
| Q4 | 0.1126 | 0.1324 | 0.2731 | 0.2470 |
| Q5 (Most) | 0.0278 | 0.0288 | 0.3813 | 0.5183 |
| Instability Quintiles |  |  |  |  |
| Q1 (Least) | 0.0906 | 0.0446 | 0.1998 | 0.0333 |
| Q2 | 0.1249 | 0.1088 | 0.1066 | 0.0625 |
| Q3 | 0.2102 | 0.1898 | 0.0976 | 0.1300 |
| Q4 | 0.3075 | 0.3238 | 0.1501 | 0.3233 |
| Q5 (Most) | 0.2668 | 0.3330 | 0.4459 | 0.4509 |

**Supplementary Table S3.** Continued.

| Indicator Variables | Class 1 | Class 2 | Class 3 | Class 4 |
| --- | --- | --- | --- | --- |
| Ethnic Concentration Quintiles |  |  |  |  |
| Q1 (Least) | 0.0166 | 0.0853 | 0.0049 | 0.0007 |
| Q2 | 0.1036 | 0.2080 | 0.0013 | 0.0048 |
| Q3 | 0.4189 | 0.3444 | 0.0045 | 0.0335 |
| Q4 | 0.4538 | 0.3070 | 0.2119 | 0.2397 |
| Q5 (Most) | 0.0071 | 0.0552 | 0.7775 | 0.7213 |
| Dependency Quintiles |  |  |  |  |
| Q1 (Least) | 0.2807 | 0.2273 | 0.5042 | 0.2923 |
| Q2 | 0.3021 | 0.2651 | 0.2457 | 0.2671 |
| Q3 | 0.2095 | 0.2121 | 0.1200 | 0.2109 |
| Q4 | 0.1177 | 0.1616 | 0.0808 | 0.1414 |
| Q5 (Most) | 0.0900 | 0.1340 | 0.0493 | 0.0883 |
| Environmental exposures |  |  |  |  |
| Environmental tobacco smoke exposure | 0.0819 | 0.0495 | 0.1636 | 0.2067 |
| Nitrogen dioxide (NO_2_) |  |  |  |  |
| Q1 | 0.5040 | 0.0042 | 0.4220 | 0.0029 |
| Q2 | 0.4307 | 0.0803 | 0.3776 | 0.0468 |
| Q3 | 0.0641 | 0.4707 | 0.1724 | 0.3136 |
| Q4 | 0.0011 | 0.4447 | 0.0280 | 0.6366 |
| Ozone (O_3_) |  |  |  |  |
| Q1 | 0.0000 | 0.5685 | 0.0022 | 0.5927 |
| Q2 | 0.3090 | 0.1833 | 0.2618 | 0.2215 |
| Q3 | 0.3786 | 0.1135 | 0.3414 | 0.0998 |
| Q4 | 0.3124 | 0.1347 | 0.3946 | 0.0860 |
| Fine particulate matter ≤2.5 microns in width (PM_2.5_) |  |  |  |  |
| Q1 | 0.4599 | 0.0114 | 0.4536 | 0.0508 |
| Q2 | 0.3231 | 0.1319 | 0.3220 | 0.1723 |
| Q3 | 0.1871 | 0.3117 | 0.1712 | 0.3607 |
| Q4 | 0.0299 | 0.5450 | 0.0532 | 0.4162 |
| Normalized Difference Vegetation Index (NDVI) |  |  |  |  |
| Q1 | 0.1366 | 0.1697 | 0.3530 | 0.4104 |
| Q2 | 0.1867 | 0.2222 | 0.3122 | 0.3188 |
| Q3 | 0.2860 | 0.2948 | 0.2007 | 0.1817 |
| Q4 | 0.3907 | 0.3132 | 0.1341 | 0.0891 |

**Supplementary Figure S1.** Cohort selection flow chart.


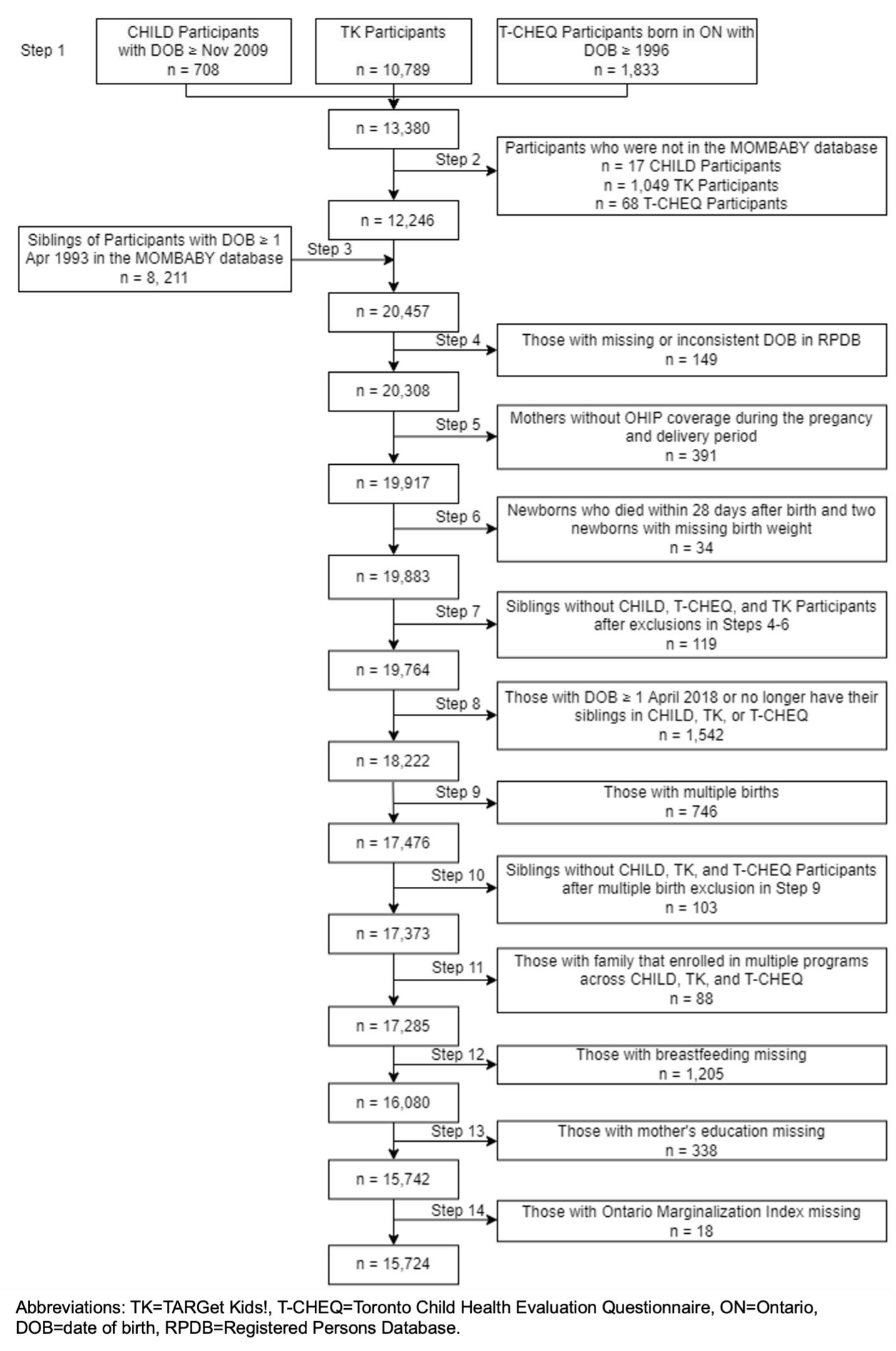


**Supplementary Figure S2.** Radar plots of Latent Class Analysis of 16 indicators in three domains.


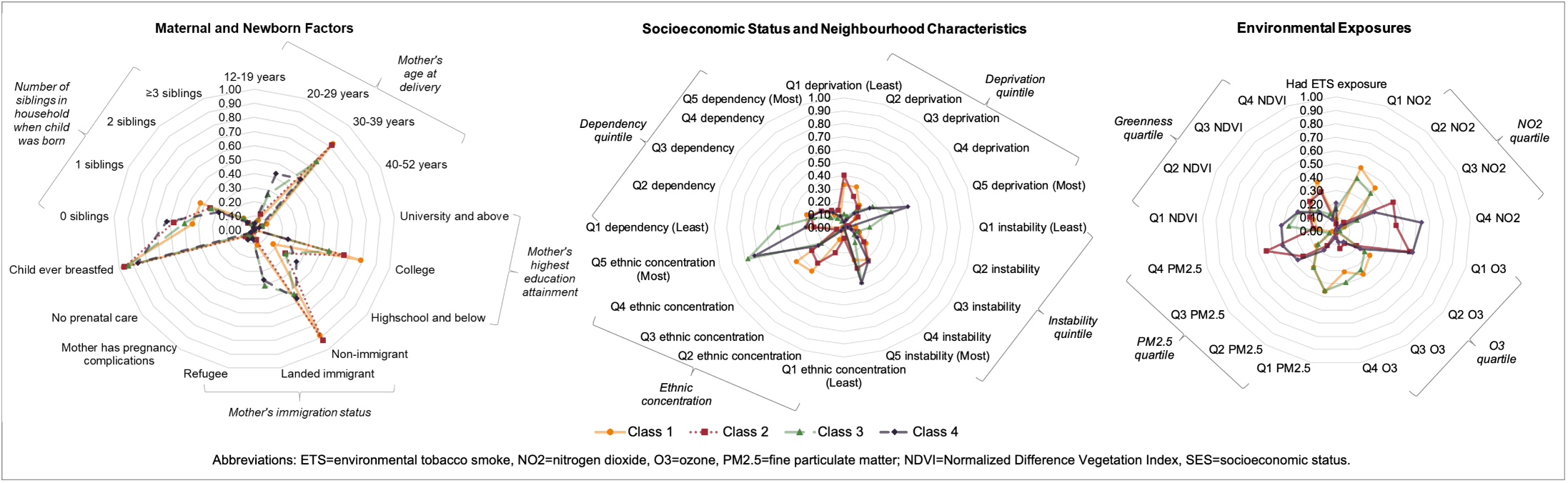


Latent class identification: **Class 1: *mothers in their 30s-40s*** with university or above education, non-immigrants, who likely had one or two children, lived in high SES neighborhoods with good air quality and greenspace; **Class 2: *mothers who were > 30s*** with university or above education, non-immigrants, who likely had a single child, lived in a high SES neighborhood, but with relatively poorer air quality and lower amount of greenspace; **Class 3: *mothers in their 30s*** with university or above education, likely a landed immigrant (or a refugee), with one or more children, lived in average SES neighborhoods with relatively good air quality and a good amount of greenspace; **Class 4:** ***mothers in their 20s*** with high school to college education, likely a landed immigrant (or a refugee) and with a single child, lived in low SES neighborhoods with high traffic-related air pollution and lower amount of greenspace.
